# 18F-fluoride PET/MR in cardiac amyloid: A comparison study with aortic stenosis and age- and sex-matched controls

**DOI:** 10.1007/s12350-020-02356-1

**Published:** 2020-09-30

**Authors:** Jack P. M. Andrews, Maria Giovanni Trivieri, Russell Everett, Nicholas Spath, Gillian MacNaught, Alastair J. Moss, Mhairi K. Doris, Tania Pawade, Edwin J. R. van Beek, Christophe Lucatelli, David E. Newby, Philip Robson, Zahi A. Fayad, Marc R. Dweck

**Affiliations:** 1 British Heart Foundation Centre for Cardiovascular Science, University of Edinburgh, Room SU.305, Chancellor’s building, 51 Little France Crescent, Edinburgh, EH16 4SB UK; 2 Icahn School of Medicine at Mount Sinai, New York, NY USA; 3 BioMedical Engineering and Imaging Institute, New York, NY USA; 4 Edinburgh Imaging, Queen’s Medical Research Institute University of Edinburgh, Edinburgh, UK

**Keywords:** PET, PET/MR, 18F-fluoride, Amyloid, Aortic stenosis, CMR

## Abstract

**Objectives:**

Cardiac MR is widely used to diagnose cardiac amyloid, but cannot differentiate AL and ATTR subtypes: an important distinction given their differing treatments and prognoses. We used PET/MR imaging to quantify myocardial uptake of 18F-fluoride in ATTR and AL amyloid patients, as well as participants with aortic stenosis and age/sex-matched controls.

**Methods:**

In this prospective multicenter study, patients were recruited in Edinburgh and New York and underwent 18F-fluoride PET/MR imaging. Standardized volumes of interest were drawn in the septum and areas of late gadolinium enhancement to derive myocardial standardized uptake values (SUV) and tissue-to-background ratio (TBR_MEAN_) after correction for blood pool activity in the right atrium.

**Results:**

53 patients were scanned: 18 with cardiac amyloid (10 ATTR and 8 AL), 13 controls, and 22 with aortic stenosis. No differences in myocardial TBR values were observed between participants scanned in Edinburgh and New York. Mean myocardial TBR_MEAN_ values in ATTR amyloid (1.13 ± 0.16) were higher than controls (0.84 ± 0.11, *P* = .0006), aortic stenosis (0.73 ± 0.12, *P* < .0001), and those with AL amyloid (0.96 ± 0.08, *P* = .01). TBR_MEAN_ values within areas of late gadolinium enhancement provided discrimination between patients with ATTR (1.36 ± 0.23) and all other groups (e.g., AL [1.06 ± 0.07, *P* = .003]). A TBR_MEAN_ threshold >1.14 in areas of LGE demonstrated 100% sensitivity (CI 72.25 to 100%) and 100% specificity (CI 67.56 to 100%) for ATTR compared to AL amyloid (AUC 1, *P* = .0004).

**Conclusion:**

Quantitative 18F-fluoride PET/MR imaging can distinguish ATTR amyloid from other similar phenotypes and holds promise in improving the diagnosis of this condition.

**Electronic supplementary material:**

The online version of this article (10.1007/s12350-020-02356-1) contains supplementary material, which is available to authorized users.

## Introduction

Systemic amyloidosis represents a spectrum of conditions characterized by disordered protein folding and fibrils formation. The two predominant forms affecting the heart are amyloid light chain (AL) and amyloidosis transthyretin (ATTR; hereditary or wild-type).^1^ Differentiation between these two forms is important because they are associated with very different prognoses but most importantly different treatment strategies.^2^ AL amyloid is associated with a poor outcome but may be amenable to treatment with chemotherapy.^3^ In contrast, ATTR amyloid is associated with a better prognosis and is potentially responsive to novel therapies that have been developed to reduce TTR protein aggregation.^4^

The diagnosis of cardiac amyloidosis is challenging. Echocardiography is frequently unable to differentiate amyloid from other forms of left ventricular hypertrophy such as that from aortic stenosis. Furthermore, some studies have suggested that cardiac amyloidosis is present in 6% to 29% of patients with significant aortic stenosis.^5^–^7^ Other data have also indicated that patients with aortic stenosis and concurrent cardiac amyloidosis have an adverse prognosis despite aortic valve replacement. It is, therefore, important to identify aortic stenosis patients with co-existent amyloidosis both in terms of predicting prognosis and because it influences interventional treatment decisions.

Endomyocardial biopsy is still the gold standard to make a diagnosis of cardiac amyloid but it is invasive, may be prone to sampling error, and is associated with procedure-related risk. Alongside histology, cardiovascular magnetic resonance (CMR) is increasingly being used to aid in the diagnosis of amyloidosis based largely upon the characteristic pattern of late gadolinium enhancement and T1 mapping^8^–^11^: CMR alone cannot, however, reliably distinguish AL from ATTR subtypes. SPECT bone tracers (^99m^Tc-DPD, ^99m^Tc-HMDP and ^99m^Tc-PYP) can provide this discrimination, demonstrating increased cardiac uptake in ATTR compared to AL amyloid and control subjects, but these approaches only provide a semi-quantitative assessment of activity.^12^,^13^ Conversely, we have shown in prior work that 18F-fluoride, a PET bone tracer,^14^,^15^ might have the potential to discriminate between AL and TTR amyloid in a quantitative manner, and could allow improved discrimination and monitoring of response to therapy.^16^,^17^ Lastly, the simultaneous acquisition of 18F-fluoride-PET and MR imaging using a single co-registered scan (PET/MR) has the advantage of combining the two modalities in the assessment of patients with amyloid.

In this novel prospective multicenter study, we aim to build on the initial findings by Trivieri et al,^17^ and investigate whether 18F-fluoride PET/MR imaging can help with the diagnosis of TTR amyloidosis and differentiate patients with AL cardiac amyloid, aortic stenosis, and age/sex-matched controls.

## Methods

### Patient Recruitment

This multicenter hybrid imaging study was conducted between December 2015 and June 2018 at two sites: The British Heart Foundation centre for Cardiovascular Science at the University of Edinburgh, UK and the Icahn School of Medicine in New York, USA. All participants were older than 50 years. Patients with cardiac amyloid were recruited from outpatient clinics and inpatient wards over both sites. The diagnosis of AL or TTR amyloid was established on histological analysis of biopsy samples in all patients bar one who had Multiple Myeloma. This patient had CMR features of amyloid (included in the AL group) but did not have a tissue biopsy. Age/sex-matched subjects were recruited as a negative control group as were patients with aortic stenosis (peak aortic jet velocity of > 2.5 m/s, with no clinical suspicion of amyloid).

Exclusion criteria for all cohorts included inability to receive iodinated contrast, renal impairment (estimated glomerular filtration rate ≤ 30 mL/min/1.73 m^2^) or women of child-bearing potential.

For those recruited in Edinburgh, the study was approved by the Scottish Research Ethics Committee and the United Kingdom (UK) Administration of Radiation Substances Advisory Committee. It was performed in accordance with the Declaration of Helsinki and all patients provided written informed consent prior to any study procedures. Subjects recruited in New York had ethical and Institutional Review Board approval for the study (GCO#01-1032). All participants provided written informed consent. The study was registered on Clinicaltrials.gov (NCT03626584). We did not directly include patient and public involvement (PPI) in this study, but the patient information sheet used in the study was developed with PPI and was reviewed by a committee that includes patient representatives.

### Imaging Protocols

#### ^18^F-fluoride positron emission tomography and coronary magnetic resonance angiography

This was a multicenter PET/MR study, but where possible protocols were standardized between centers with all patients undergoing simultaneous PET and MR imaging using the same hybrid PET/MR system (Biograph mMR, Siemens Healthcare GmbH, Erlangen, Germany). The MR protocol at each site was the same including long axis cine imaging (3-chamber, 2-chamber, 4-chamber), a short axis cine stack (8 mm thickness, 1.6 mm gap) coronary magnetic resonance angiography (CMRA for accurate co-registration) performed with 0.2 mmol/kg of intravenous gadobutrol contrast (Gadovist, Bayer Pharma AG, Germany) and late gadolinium enhancement imaging 10 to 15 minute post-contrast administration. Importantly, the same radial gradient recalled echo (GRE, Siemens work-in-progress #793F) sequences was acquired for MR attenuation correction at both sites as previously described,^6^. PET imaging was performed 60 to 120 minutes post administration of 125-350MBq 18F-fluoride. List mode PET data were then reconstructed using e7tools (Siemens Healthcare) applying the radial GRE sequence (2 tissue classes: background [air and lung] and soft tissue [soft tissue and fat]).^18^,^19^ An Ordered Subsets Expectation Maximization (OSEM) algorithm with the following parameters was employed: 256 × 256 matrix, 4 iterations, 21 subsets, 5 mm Gaussian filter in Edinburgh and 344 × 344 matrix, 6 iterations, 21 subsets, 2 mm Gaussian filter in New York.

#### PET/MR image analysis

**Figure 1:**
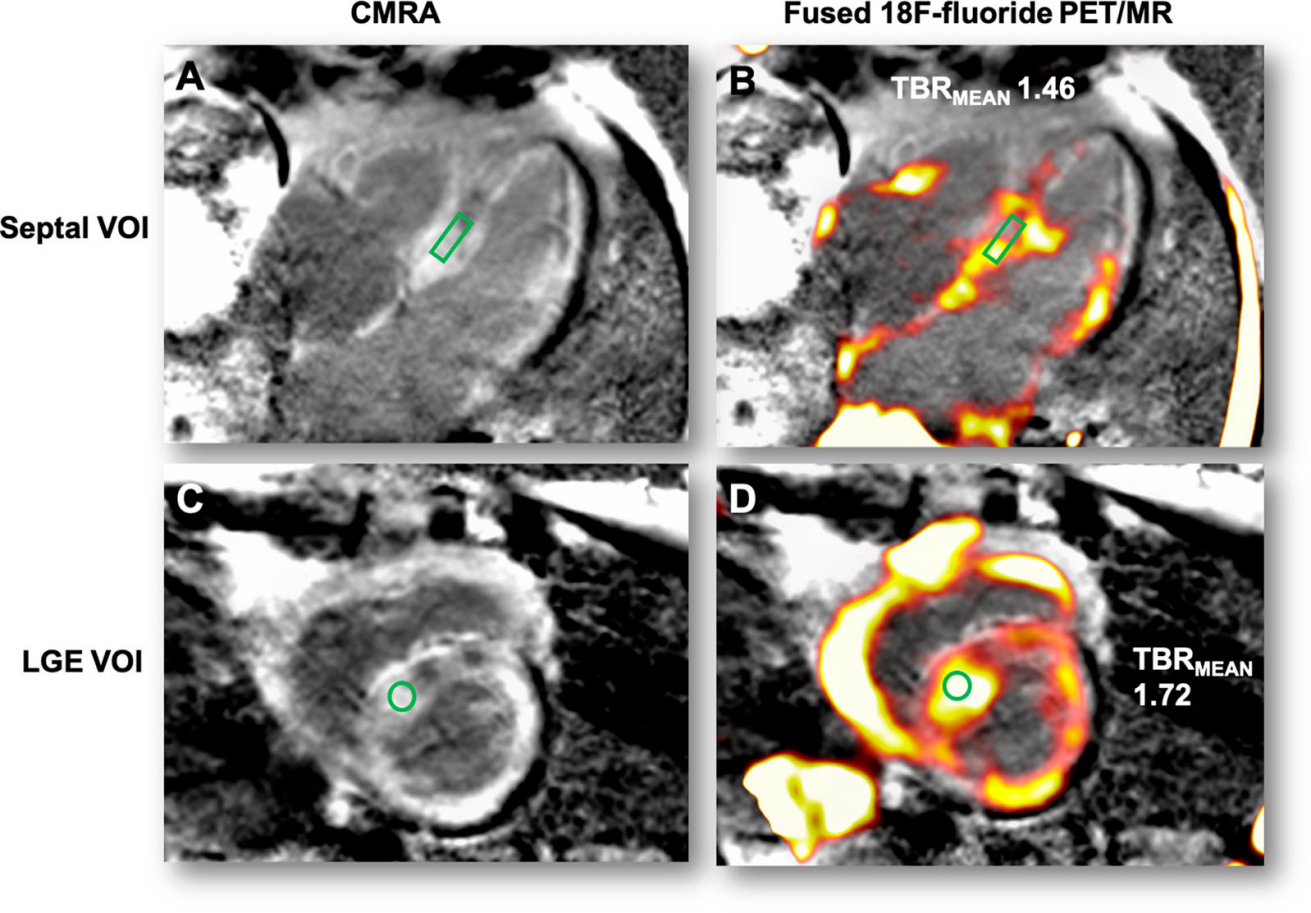
Derivation of myocardial and LGE volumes of interest. Panel **A** is a 4-chamber view with LGE showing typical myocardial nulling difficulties in a patient with TTR cardiac amyloidosis. The standardized 3 × 15 mm green cylindrical VOI is placed in the septum at the mid-ventricular level. Panel **B** shows the fused PET/MR with high uptake in the septal and lateral wall of the left ventricle and free wall of the right ventricle (VOI TBR_MEAN_ inset). Panel **C** shows the LGE image in the short axis with extensive diffuse LGE affecting most of the myocardium with the corresponding short axis view of the septal VOI. Panel **D** shows the fused PET/MR image of the same slice with diffuse uptake within the left and right ventricular myocardium. Note the intensely high uptake within the most diseased myocardial segment (within the septal VOI) and the difference in TBR_MEAN_ compared to panel **B**

All PET/MR images were analyzed at the University of Edinburgh Core Lab by two expert readers (JA, MRD). Accurate co-registration was achieved by aligning ^18^F-fluoride activity in the blood pool and ascending aorta with the corresponding anatomical structures on the CMRA.^20^ Qualitative and semi-quantitative analysis of the PET images was performed using FusionQuant software (Cedars-Sinai Medical Center, Los Angeles). Radiotracer uptake was analyzed using a standardized protocol (supplemental data). For myocardial analysis two approaches were employed. First, based on previous research showing amyloid infiltration and septal hypertrophy in 79% of ATTR cases we sampled septal uptake.^21^ This was calculated using cylinders of 3 mm radius and 15 mm length set within the septum at mid cavity level on the co-registered co-axial image to generate volumes of interest (VOI, Figure 1, supplementary protocol). Standardized uptake values (SUV_MEAN_ and SUV_MAX_) were calculated for these septal VOIs and corrected for blood pool activity (measured in the right atrium)^22^ to provide tissue-to-background ratio (TBR_MEAN_ and TBR_MAX_). Second, in patients with cardiac amyloid ^I8^F-fluoride uptake was assessed in areas of myocardial LGE. Equal sized volumes of interest (VOI) were placed within areas of LGE demonstrating the greatest visual uptake on short axis slices with SUV_MEAN_ and TBR_MEAN_ values recorded (Figure 1D).

Throughout the study TBR values were used in preference for comparison. In principle the correction of tissue uptake for blood pool measurements made on the same scan adjusts for potential differences in PET values acquired at different scanners. In order to test this principle, we compared TBR_MEAN_ values for both healthy volunteers and amyloid patients imaged in Edinburgh to the equivalent subjects imaged in New York.

### Statistical Analysis

All statistical analyses were performed using GraphPad Prism Version 8.0. A two-sided *P* < 0.05 was considered statistically significant. The distribution of all continuous variables was assessed using the Shapiro-Wilk test, which were presented using mean ± standard deviation or median [interquartile range]. Comparisons between groups were performed using the two-sample *t* test, Mann-Whitney test or ordinary one-way ANOVA as appropriate. Receiver operating characteristic (ROC) curves were generated to determine area under curve (AUC) and perform sensitivity and specificity analyses. We presented all categorical variables as percentages.

## Results

53 patients (33 in Edinburgh and 20 in New York) were scanned and completed the study protocol without complication: 13 controls, 18 with cardiac amyloid (10 ATTR and 8 AL), and 22 aortic stenosis (Table 1).

**Table 1:** Participant clinical, radiological, and CMR characteristics

Clinical characteristics	Age- and sex-matched controls (n = 13)	Aortic stenosis (n = 22)	Amyloid (n = 18)
Age	65 ± 15	76 ± 8	70 ± 9
Male	8/13 (62%)	15/22 (68%)	14/18 (78%)
BMI (kg/m^2^)	26.0 ± 3.0	28.6 ± 4.2	24.4 ± 5.1
Smoking (ex or current)	3/13(23%)	9/22 (41%)	8/18 (44%)
Hypertension	4/13 (31%)	13/22 (59%)	11/18 (61%)
Hyperlipidaemia	4/13 (31%)	11/22 (50%)	8/18 (44%)
Diabetes	1/13 (8%)	4/22 (18%)	5/18 (28%)
Previous myocardial Infarction	0/13 (0%)	2/22 (9%)	2/18 (11%)
Previous PCI	0/13(0%)	3/22 (14%)	3/18 (17%)
Imaged in Edinburgh	6	22	5
Administered dose ^18^F-Fluoride (MBq)	308 ± 80	201 ± 58	333 ± 91
PET/MR injection-to-scan interval (mins)	50 ± 18	80 ± 31	55 ± 22
Body surface area (m^2^)	1.91 ± 0.20	1.96 ± 0.17	1.86 ± 0.26
LVEDi	72 ± 20	76 ± 24	72 ± 21
LVESVi	18 ± 5	26 ± 23	31 ± 25
LVSVi	55 ± 17	52 ± 11	40 ± 11
Ejection Fraction (%)	75 ± 6	70 ± 17	60 ± 18
LVMi	64 ± 8	100 ± 26	101 ± 36

*BMI*, body mass index; *PCI*, percutaneous coronary intervention; *PET/MR*, positron emission tomography/magnetic resonance; *CMR*, cardiac magnetic resonance; *LVEDi*, left ventricular end diastolic volume indexed; *LVESVi*, left ventricular end systolic volume indexed; *LVSVi*, left ventricular stroke volume indexed; *LVMi*, left ventricular mass indexed

Despite differences in scanning protocol and PET reconstruction between the two sites, there was no significant difference in myocardial TBR_MEAN_ values in either the control subject nor the amyloid patients (Table 2).

**Table 2:** Comparison of TBR_MEAN_ values between centers

	Edinburgh TBR_MEAN_	New York TBR_MEAN_	*P* value
Age/sex-matched controls	0.84 ± 0.08	0.88 ± 0.11	*P* = .53
Amyloid	1.11 ± 0.21	1.03 ± 0.04	*P* = .37

*TBR*, tissue-to-background ratio

### Septal Myocardial 18F-Fluoride Activity

CMR was normal in all controls (Table 1). Myocardial 18F-fluoride uptake in their normal myocardium was lower than blood pool (septal TBR_MEAN_ 0.86 ± 0.10 *P* < .0001), allowing the cavity of the blood pool in the left and right ventricles to be delineated and facilitating accurate co-registration of the MR angiogram and PET datasets in 3 dimensions. Patients with aortic stenosis had left ventricular hypertrophy with elevated LV mass index (100 ± 26 g/m^2^) but normal ejection fraction (69 ± 17 %). 18F-flouride uptake was again lower than blood pool (TBR_MEAN_ 0.73 ± 0.12, *P* = .03).

18 patients with cardiac amyloidosis were scanned: 10 (56%) with ATTR and 8 (44%) with AL cardiac amyloid. Mean myocardial mass was elevated at 101 ± 37g/m^2^ with mean ejection fraction of 60 ± 18%. 15/18 (83%) patients had a pattern of diffuse generalized pattern of LGE on CMR (8 with ATTR and 7 with AL). Two patients (1 TTR and 1 AL) had focal LGE, while 1 (TTR) had no appreciable LGE on CMR.

Increased septal 18F-fluoride activity was observed in patients with ATTR amyloid (1.13 ± 0.16) compared to healthy volunteers (0.86 ± 0.10, *P* = .0002) and patients with both AL amyloid (0.95 ± 0.08, *P* = .01) and aortic stenosis (0.73 ± 0.12, *P* < .0001; Table 3, Figure 3A). There was no increase in septal 18F-fluoride uptake in patients with AL amyloidosis compared to controls (0.95 ± 0.08, *P* = .54).

**Table 3:** Comparison of TBR_MEAN_ between groups

	Septal TBR_MEAN_	LGE TBR_MEAN_	*P* value
Age/sex-matched controls	0.86 ± 0.10	–	–
Aortic stenosis	0.73 ± 0.12	–	–
AL amyloid	0.96 ± 0.08	1.06 ± 0.06	*P* = .01
ATTR amyloid	1.13 ± 0.16	1.39 ± 0.23	*P* = .02

TBR, tissue-to-background ratio; *LGE*, late gadolinium enhancement; *AL*, amyloid light chain; *TTR*, transthyretin

### Myocardial 18F-Fluoride Activity in Areas of Late Gadolinium Enhancement

**Figure 2:**
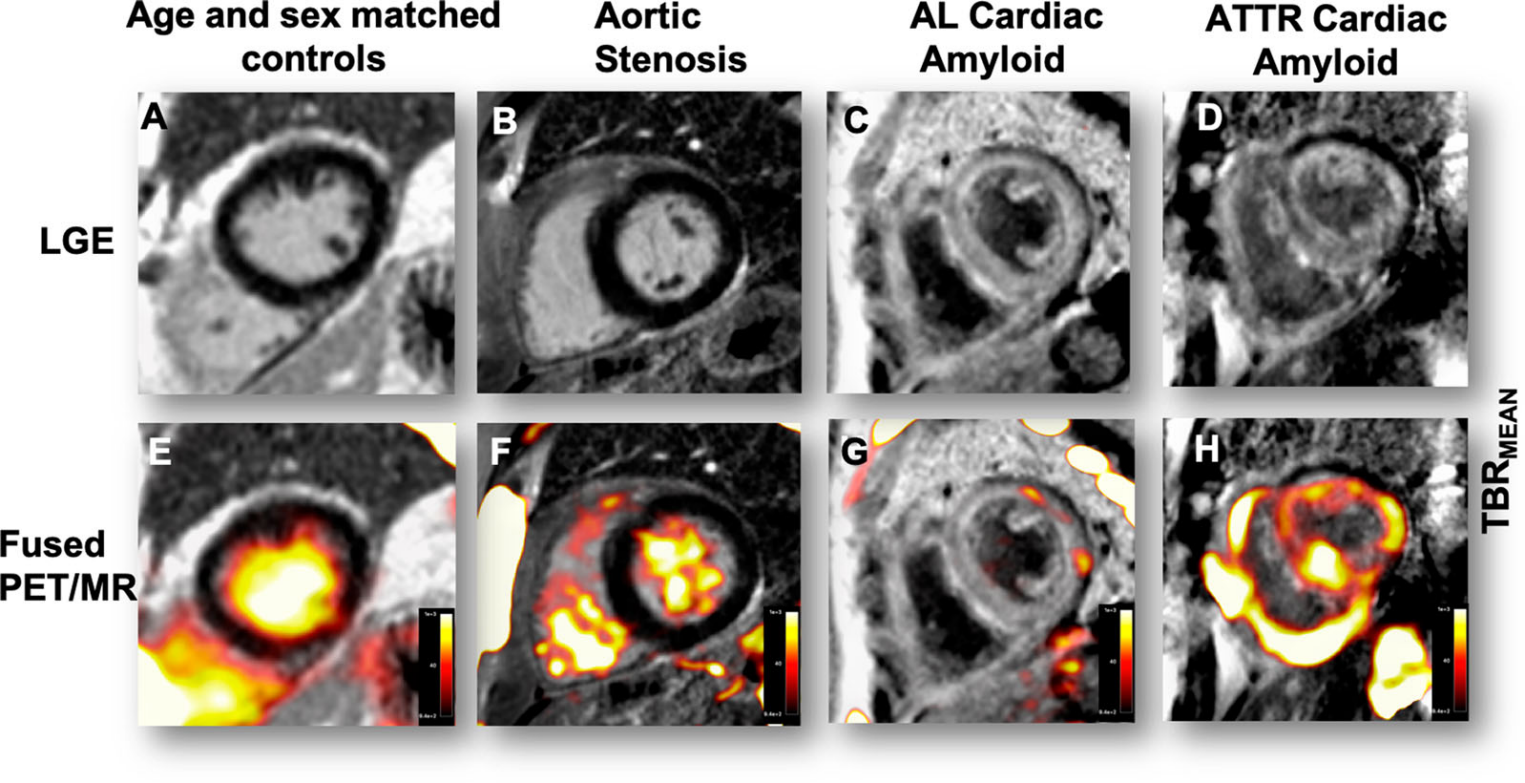
Patterns of 18F-fluoride uptake between cohorts. Columns represent each cohort and rows imaging modality in the short axis view. Panel **A** shows a delayed enhanced image of a control subject with normal myocardial mass and no LGE. The corresponding fused PET/MR image (**E**) shows uptake only in the blood pool. Panel **B** is a patient with aortic stenosis and elevated LV mass. Note the absence of myocardial 18F-fluoride uptake on panel **F** and similar to the healthy control, uptake is greater in the blood pool than myocardium. Panel **C** shows a patient with AL amyloid displaying the characteristic myocardial nulling difficulties with LGE found in cardiac amyloidosis. Panel **G** shows patchy lateral wall uptake greater than the blood pool. Panel **D** shows similar LGE findings, but this time in TTR amyloid. Note the striking and extensive biventricular uptake in panel **H**, much greater than the blood pool and what was seen in AL

**Figure 3:**
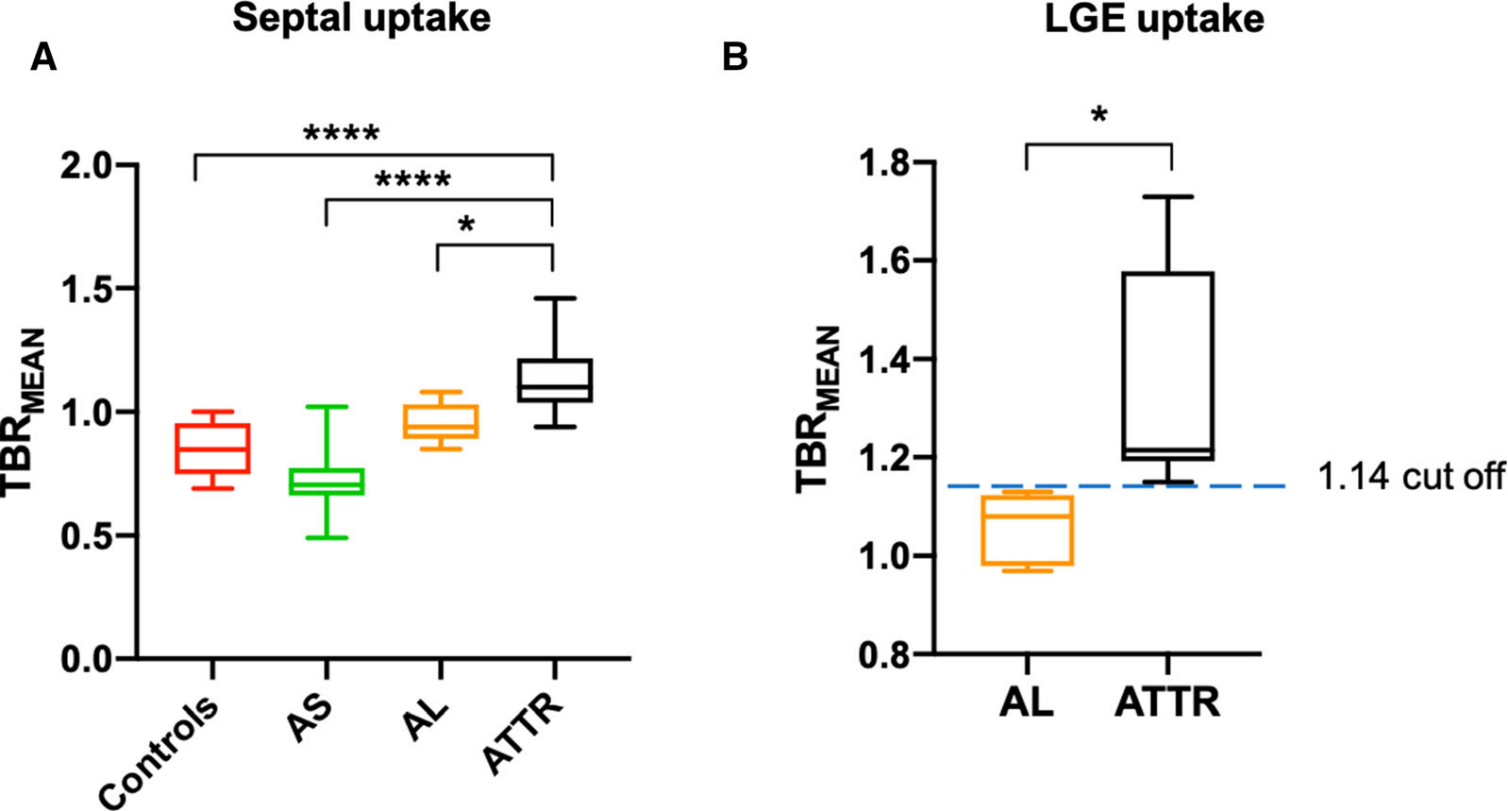
Comparison of myocardial TBR_MEAN_ across all cohorts and both subtypes of cardiac amyloid. TBR_MEAN_ in graph A allows differentiation between ATTR and all cohorts. Further differentiation between amyloid subtypes can be appreciated in graph B with TTR displaying greater uptake than AL. Moreover, within areas of LGE, a cut-off value of > 1.14 gives 100% sensitivity and 100% specificity to detect ATTR over AL. Ordinary one-way ANOVA and unpaired *t* test. *< 0.05, **< 0.01, ***< 0.001, ****< 0.0001

In the ATTR patients the most intense myocardial 18F-fluoride uptake was observed in areas of LGE (Figure 2). Indeed, TBR values were 35% higher in these LGE regions compared to other areas of ‘normal’ myocardium in the same patients (1.39 ± 0.23 vs 0.90 ± 0.23, *P* < .0002). Moreover 18F-fluoride TBR values in areas of LGE provided excellent discrimination between patients with TTR and AL amyloid (Figure 3B) with a TBR_MEAN_ threshold >1.14 providing 100% sensitivity (CI 72.25 to 100%) and 100% specificity (CI 67.56 to 100%) for TTR amyloid (AUC 1, *P* = .0004).

## Discussion

In the first multicenter study of its kind, we have used hybrid PET/MR to investigate myocardial 18F-fluoride activity in patients with TTR amyloid, demonstrating increased uptake compared to age- and sex-matched controls and patients with both aortic stenosis and AL amyloid (Table 3 + Figure 3A). Indeed, when sampled within areas of myocardial disease, a TBR_MEAN_ threshold of 1.14 provided perfect discrimination between patients with ATTR and these other conditions. Our findings are in keeping with our own and recently published work by other groups.^17^,^23^,^24^

The diagnosis and treatment of cardiac amyloidosis has advanced markedly over the last 10 years. AL amyloid can now be treated with disease modifying novel chemotherapeutic agents^25^,^26^, while contemporary TTR therapies target suppression of TTR expression and stabilization of transthyretin, improving patients outcomes.^4^ The accurate diagnosis of TTR amyloid is, therefore, of major importance. However, the mechanism of benefit of these drugs on the myocardium is not well understood and importantly improvements in myocardial performance after therapy are frequently absent. Thus, there is a need for novel imaging techniques that can elucidate the effects of these novel drugs on the myocardium and that can track disease progression and response to therapy. Each of the currently available imaging techniques has well recognized limitations in these regards. CMR is the reference standard imaging test for the diagnosis of cardiac amyloid, but cannot differentiate between AL and TTR amyloid. SPECT bone tracers demonstrate increased uptake in TTR amyloid but provide only imperfect discrimination from AL and semi-quantitative measurements poorly suited to detecting treatment response. While PET imaging with amyloid specific tracer offers fully quantitative assessments of amyloid burden, these tracers are expensive and do not fully differentiate between AL and TTR amyloid.

18F-fluoride PET/MR appears to provide a solution to some of these problems and, therefore, holds promise in the diagnosis and monitoring of patients with TTR amyloid. This approach combines the accurate diagnosis of cardiac amyloid on the CMR with clear discrimination of biopsy proven AL and TTR amyloid on the 18F-fluoride PET in a single scan. Hybrid imaging also facilitates accurate co-registration and comparison of the CMR and PET images. This is an important advantage as it allows regions of interest to be accurately drawn in specific regions of the myocardium including areas of late gadolinium enhancement wherein TBR values provide the greatest discriminatory information. The value of hybrid imaging will be further explored in the upcoming ‘I-CARE’ study which will evaluate the incremental benefit of tissue imaging with CMR to 18F-fluoride PET/CT. Finally, although theoretical at present, the fully quantitative nature of PET could potentially provide an assessment of TTR burden that could be used to track disease progression and/or treatment responses to the array of new therapies being developed for this condition. Indeed, PET imaging has been used in a similar fashion in other contexts assessing treatments responses to both atherosclerosis and aortic stenosis (SALTIRE 2 clinicaltrials.gov, NCT02132026).

While patterns of late gadolinium enhancement tend to differ between patients with ATTR and aortic stenosis, we have also demonstrated that 18F-fluoride PET/MR can too provide excellent discrimination between ATTR and aortic stenosis. This too is becoming an increasingly important and challenging clinical distinction. Recent studies have suggested that up to 12% patients with significant aortic stenosis undergoing surgical AVR have co-existent cardiac amyloid.^5^,^6^ Furthermore, 16% of patients referred for TAVI (transcatheter aortic valve insertion) had uptake (Tc-99m-PYP) consistent with ATTR, 62% of whom met the criteria for low-flow, low-gradient severe aortic stenosis.^27^ Treatments for aortic stenosis and amyloid differ;^3^,^4^ in particular patients with ATTR may not be best served with transcutaneous or surgical aortic valve replacement.

Our observations in the healthy control group are also of importance. A key advantage of 18F-fluoride imaging in the heart is the low myocardial activity, with uptake values 80% of that observed in the blood pool. This has several implications that facilitate the wider assessment of 18F-fluoride PET as a marker of disease activity in the coronary arteries and heart valves.^18^,^28^–^31^ First it allows accurate co-registration of the blood pool signal on the PET with the cardiac chambers on MR angiography. Second it means that even relatively low 18-fluoride uptake in the coronary arteries can be differentiated from blood pool and the activity in the adjacent myocardium.

## Limitations

To our knowledge, this is the largest multicenter study investigating myocardial 18F-fluoride uptake in cardiac amyloid and while there is an important need for multicenter cardiovascular PET studies, this approach comes with inherent challenges and limitations. These include variation in the injected activity of 18F-fluoride, reconstruction parameters, and between scanner differences in uptake measurement values. Despite these potential problems we have demonstrated that TBR values appear to correct for many of these between center variations with no difference in TBR values measured in equivalent patients imaged at the two centers. Future multicenter cardiovascular PET studies should, therefore, be encouraged in the knowledge that many between center differences can be overcome.

## Conclusion

We have demonstrated that myocardial 18F-fluoride PET uptake is increased in patients with TTR amyloid, with TBR values providing quantification and clear discrimination from other similar conditions such as AL amyloid and aortic stenosis. This hybrid imaging technique may, therefore, help in the diagnosis of ATTR amyloid.

## New Knowledge Gained

- We identified that ATTR binds 18F-fluoride more avidly than AL and the negative control groups
- We present 18F-fluoride PET/MR as a hybrid imaging technique able to distinguish between amyloid subtypes, phenotypically similar patients with aortic stenosis and age/sex-matched controls.
- This may improve our ability as clinicians to make the correct diagnosis and offer appropriate disease specific treatment.

## Electronic supplementary material

Below is the link to the electronic supplementary material.

Electronic supplementary material 1 (DOCX 16 kb)

Electronic supplementary material 2 (PPTX 2118 kb)

Electronic supplementary material 3 (M4A 12773 kb)

## Abbreviations

AL: Amyloid light chain
ATTR: Amyloid transthyretin
CMR: Cardiac magnetic resonance
SPECT: Single photon emission computerized tomography
^99m^Tc-DPD: ^99^Technicium- diphosphono-propanedicarboxylic acid
^99m^Tc-HMDP: ^99^Technicium- hydroxymethylene diphosphonate
^99m^Tc-PYP: ^99^Technicium- pyrophosphate
PET/MR: Positron emission tomography/magnetic resonance
MGUS: Monoclonal gammopathy of unknown significance
CMRA: Cardiac magnetic resonance angiography
GRE: Gradient recalled echo
PET: Positron emission tomography
OSEM: Ordered subsets maximization
MBq: Megabecquerels
VOI: Volume of interest
SUV_MEAN_: Mean standardized uptake value
SUV_MAX_: Max standardized uptake value
TBR_MEAN_: Mean target to background ratio
TBR_MAX_: Max target to background ratio
LGE: Late gadolinium enhancement
ANOVA: Analysis of variance
ROC: Receiver operating characteristic
AUC: Area under the curve
TAVI: Transfemoral aortic valve insertion
