# Supplementary material for: 18F-fluoride PET/MR in cardiac amyloid: A comparison study with aortic stenosis and age- and sex-matched controls
Source: J Nucl Cardiol. 2020 Sep 30;29(2):741–9. doi: 10.1007/s12350-020-02356-1 (PMC8993737; doi:10.1007/s12350-020-02356-1)
Supplement: Supplementary file 1 — Electronic supplementary material 1 (DOCX 16 kb) [file 12350_2020_2356_MOESM1_ESM.docx]

**Supplemental table 1.** Comparison of TBR_MAX_ values

|  | TTR Amyloid | AL  Amyloid | P value |
| --- | --- | --- | --- |
| Septal TBR_MAX_ | 1.24±0.17 | 1.07±0.10 | p=0.02 |
| LGE TBR_MAX_ | 1.49±0.31 | 1.16±0.12 | p=0.01 |

**PET/MR myocardial Analysis Protocol**

**Version 1.0, July 2019**

Start FusionQuant software

Load MR coronary angiogram sequence as “background”

Load MRAC-corrected PET data as “overlay”:

- Gate 1: NYC radial GRE VIBE

1. Re-orientate views to show 4Ch, 2Ch and short axis at mid-ventricle level, centre image on LV cavity. Keeping in same 4Ch plane:

Draw spherical ROI in middle of RA cavity (8mm radius, approx. 2cm^3^ volume)

Record in both gates;

**- RA background SUV mean**

1. On the short axis view at mid ventricular level place 3mm x 15mm cyclinder within interventricular septum

**- Record myocardial SUV_MEAN_ and SUV_MAX_**

1. On the short axis view place circular VOI (40mm^3^) within area of greatest visual uptake of 18F-fluoride within LGE

**- Record myocardial SUV_MEAN_ and SUV_MAX_**

1. Save contours within patient study folder
