# Supplementary material for: 18F-fluoride PET/MR in cardiac amyloid: A comparison study with aortic stenosis and age- and sex-matched controls
Source: J Nucl Cardiol. 2020 Sep 30;29(2):741–9. doi: 10.1007/s12350-020-02356-1 (PMC8993737; doi:10.1007/s12350-020-02356-1)
Supplement: Supplementary file 2 — Electronic supplementary material 2 (PPTX 2118 kb) [file 12350_2020_2356_MOESM2_ESM.pptx]

## Slide 1
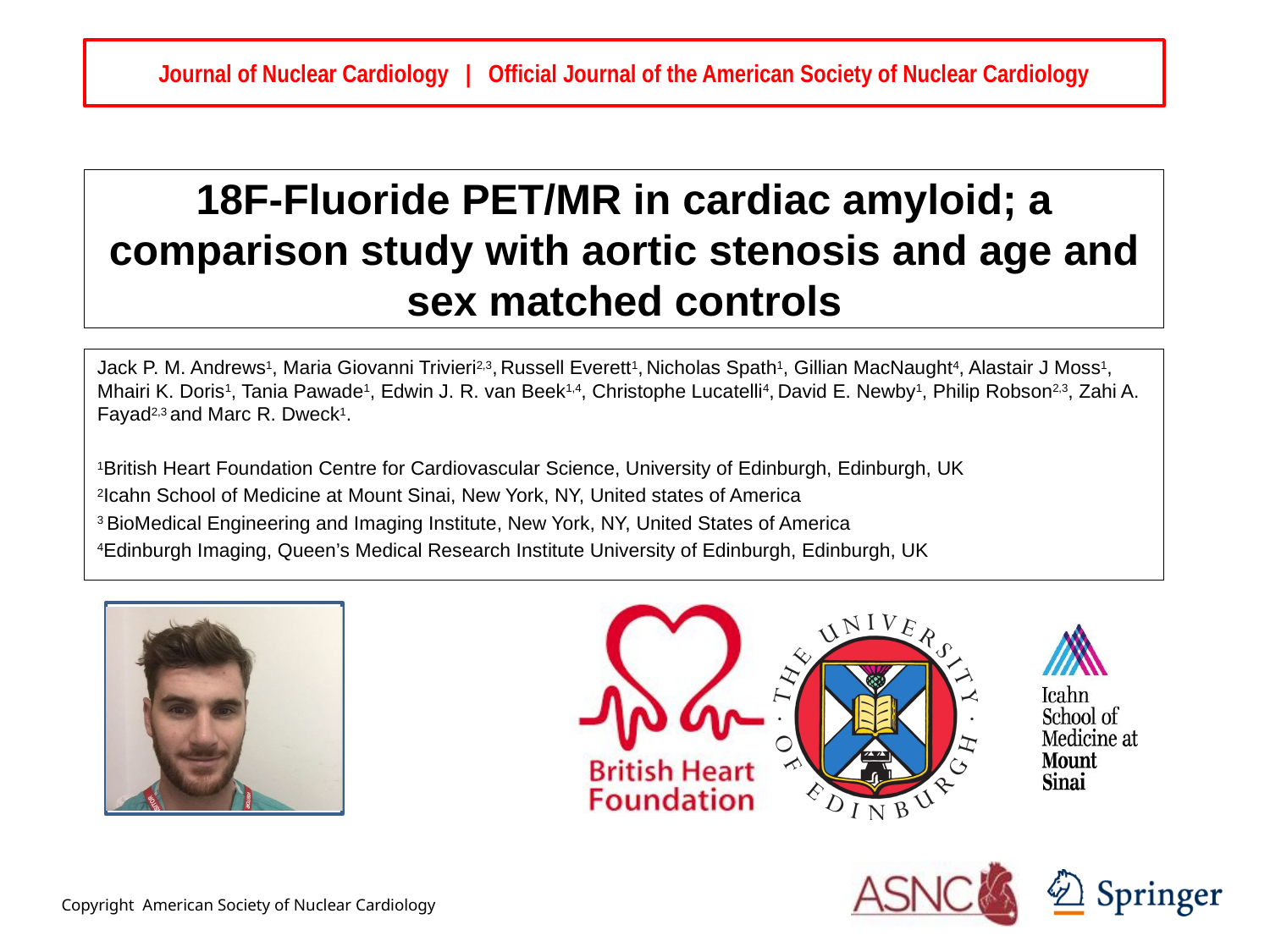

Journal of Nuclear Cardiology | Official Journal of the American Society of Nuclear Cardiology
# 18F-Fluoride PET/MR in cardiac amyloid; a comparison study with aortic stenosis and age and sex matched controls
Jack P. M. Andrews1, Maria Giovanni Trivieri2,3, Russell Everett1, Nicholas Spath1, Gillian MacNaught4, Alastair J Moss1, Mhairi K. Doris1, Tania Pawade1, Edwin J. R. van Beek1,4, Christophe Lucatelli4, David E. Newby1, Philip Robson2,3, Zahi A. Fayad2,3 and Marc R. Dweck1.
1British Heart Foundation Centre for Cardiovascular Science, University of Edinburgh, Edinburgh, UK
2Icahn School of Medicine at Mount Sinai, New York, NY, United states of America
3 BioMedical Engineering and Imaging Institute, New York, NY, United States of America
4Edinburgh Imaging, Queen’s Medical Research Institute University of Edinburgh, Edinburgh, UK
Copyright American Society of Nuclear Cardiology

## Slide 2
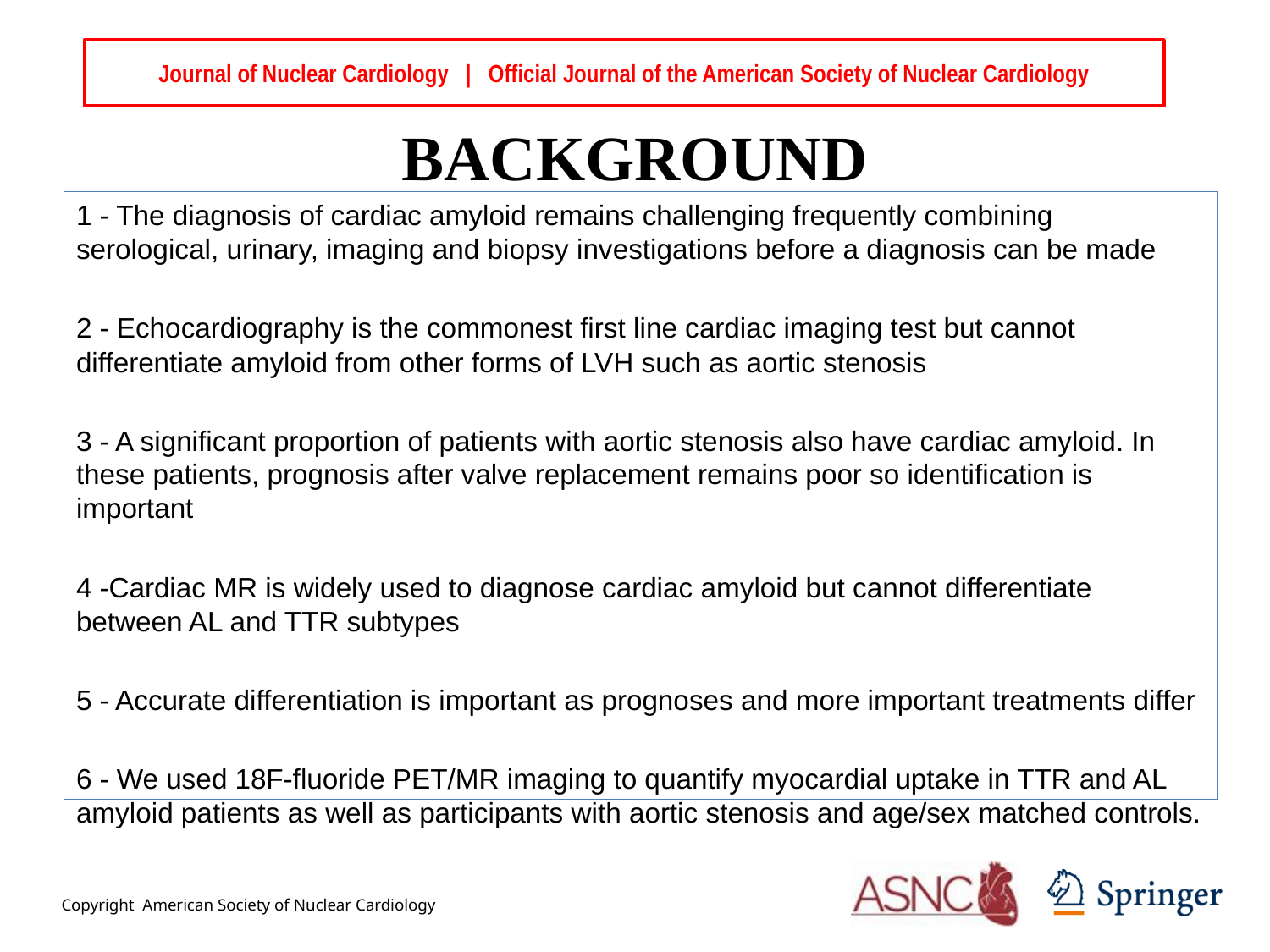

Journal of Nuclear Cardiology | Official Journal of the American Society of Nuclear Cardiology
# BACKGROUND
1 - The diagnosis of cardiac amyloid remains challenging frequently combining serological, urinary, imaging and biopsy investigations before a diagnosis can be made
2 - Echocardiography is the commonest first line cardiac imaging test but cannot differentiate amyloid from other forms of LVH such as aortic stenosis
3 - A significant proportion of patients with aortic stenosis also have cardiac amyloid. In these patients, prognosis after valve replacement remains poor so identification is important
4 -Cardiac MR is widely used to diagnose cardiac amyloid but cannot differentiate between AL and TTR subtypes
5 - Accurate differentiation is important as prognoses and more important treatments differ
6 - We used 18F-fluoride PET/MR imaging to quantify myocardial uptake in TTR and AL amyloid patients as well as participants with aortic stenosis and age/sex matched controls.
Copyright American Society of Nuclear Cardiology

## Slide 3
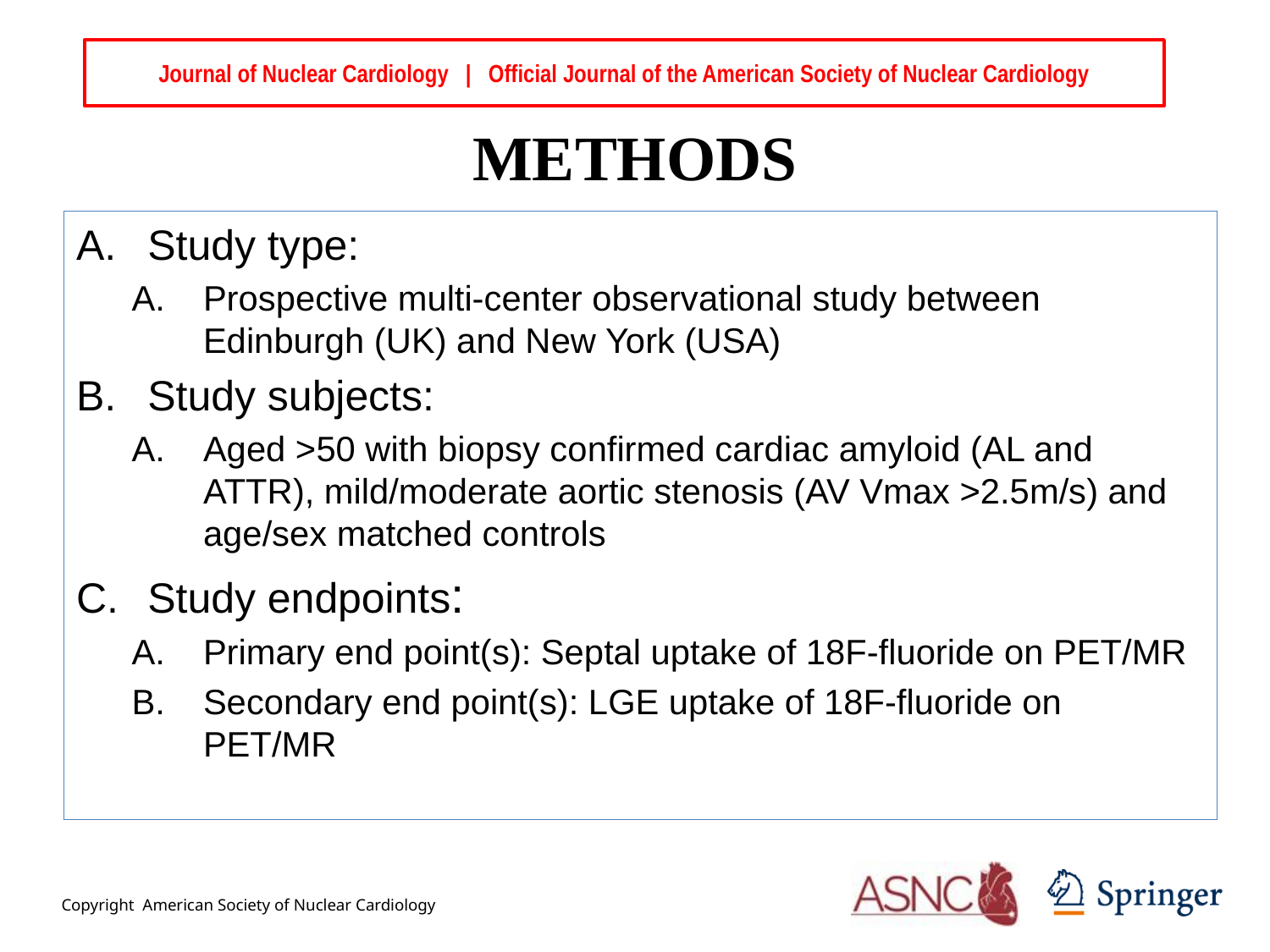

Journal of Nuclear Cardiology | Official Journal of the American Society of Nuclear Cardiology
# METHODS
Study type:
Prospective multi-center observational study between Edinburgh (UK) and New York (USA)
Study subjects:
Aged >50 with biopsy confirmed cardiac amyloid (AL and ATTR), mild/moderate aortic stenosis (AV Vmax >2.5m/s) and age/sex matched controls
Study endpoints:
Primary end point(s): Septal uptake of 18F-fluoride on PET/MR
Secondary end point(s): LGE uptake of 18F-fluoride on PET/MR
Copyright American Society of Nuclear Cardiology

## Slide 4
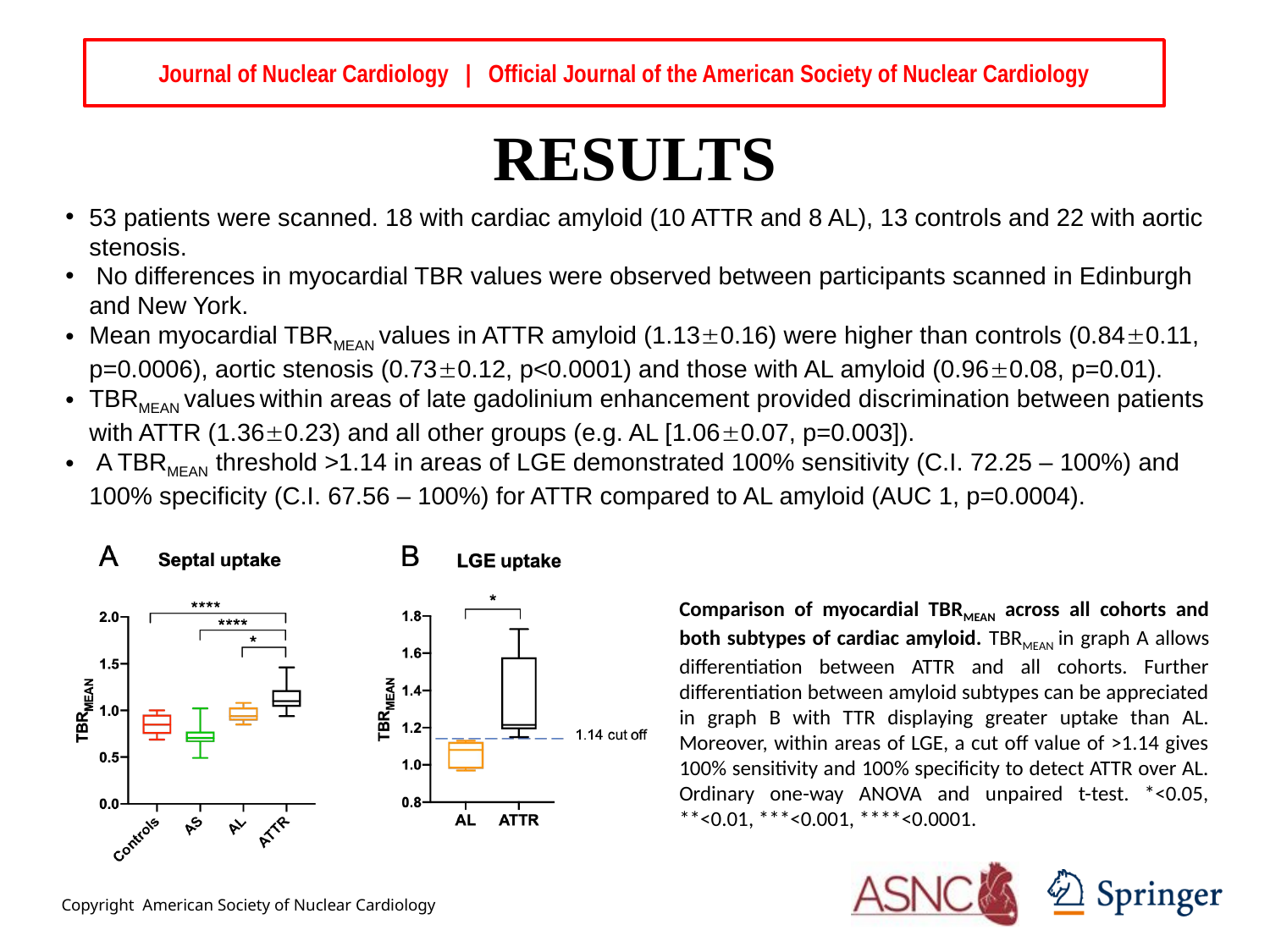

Journal of Nuclear Cardiology | Official Journal of the American Society of Nuclear Cardiology
# RESULTS
53 patients were scanned. 18 with cardiac amyloid (10 ATTR and 8 AL), 13 controls and 22 with aortic stenosis.
 No differences in myocardial TBR values were observed between participants scanned in Edinburgh and New York.
Mean myocardial TBRMEAN values in ATTR amyloid (1.13±0.16) were higher than controls (0.84±0.11, p=0.0006), aortic stenosis (0.73±0.12, p<0.0001) and those with AL amyloid (0.96±0.08, p=0.01).
TBRMEAN values within areas of late gadolinium enhancement provided discrimination between patients with ATTR (1.36±0.23) and all other groups (e.g. AL [1.06±0.07, p=0.003]).
 A TBRMEAN threshold >1.14 in areas of LGE demonstrated 100% sensitivity (C.I. 72.25 – 100%) and 100% specificity (C.I. 67.56 – 100%) for ATTR compared to AL amyloid (AUC 1, p=0.0004).
Comparison of myocardial TBRMEAN across all cohorts and both subtypes of cardiac amyloid. TBRMEAN in graph A allows differentiation between ATTR and all cohorts. Further differentiation between amyloid subtypes can be appreciated in graph B with TTR displaying greater uptake than AL. Moreover, within areas of LGE, a cut off value of >1.14 gives 100% sensitivity and 100% specificity to detect ATTR over AL. Ordinary one-way ANOVA and unpaired t-test. *<0.05, **<0.01, ***<0.001, ****<0.0001.
Copyright American Society of Nuclear Cardiology

## Slide 5
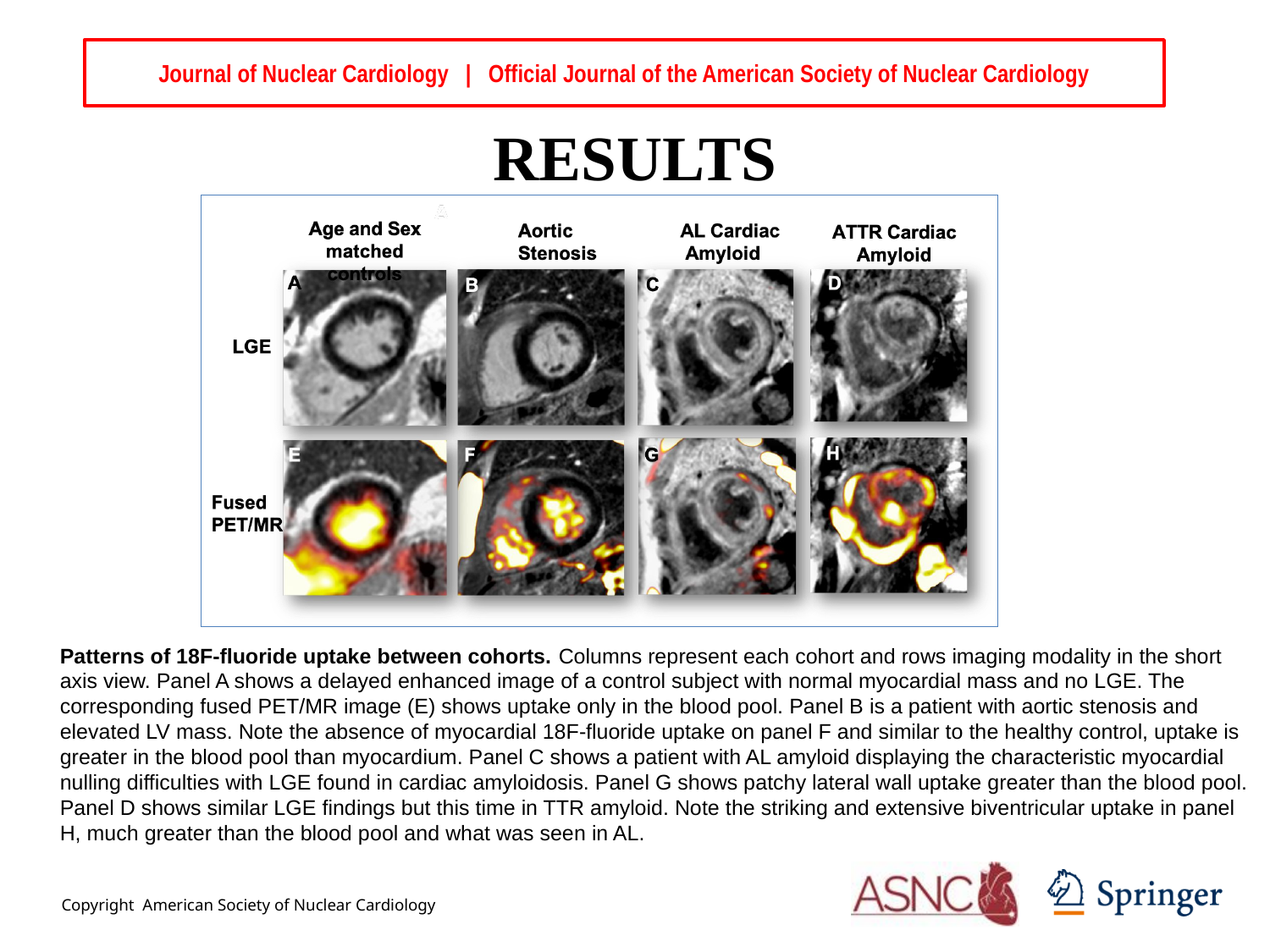

Journal of Nuclear Cardiology | Official Journal of the American Society of Nuclear Cardiology
# RESULTS
Patterns of 18F-fluoride uptake between cohorts. Columns represent each cohort and rows imaging modality in the short axis view. Panel A shows a delayed enhanced image of a control subject with normal myocardial mass and no LGE. The corresponding fused PET/MR image (E) shows uptake only in the blood pool. Panel B is a patient with aortic stenosis and elevated LV mass. Note the absence of myocardial 18F-fluoride uptake on panel F and similar to the healthy control, uptake is greater in the blood pool than myocardium. Panel C shows a patient with AL amyloid displaying the characteristic myocardial nulling difficulties with LGE found in cardiac amyloidosis. Panel G shows patchy lateral wall uptake greater than the blood pool. Panel D shows similar LGE findings but this time in TTR amyloid. Note the striking and extensive biventricular uptake in panel H, much greater than the blood pool and what was seen in AL.
Copyright American Society of Nuclear Cardiology

## Slide 6
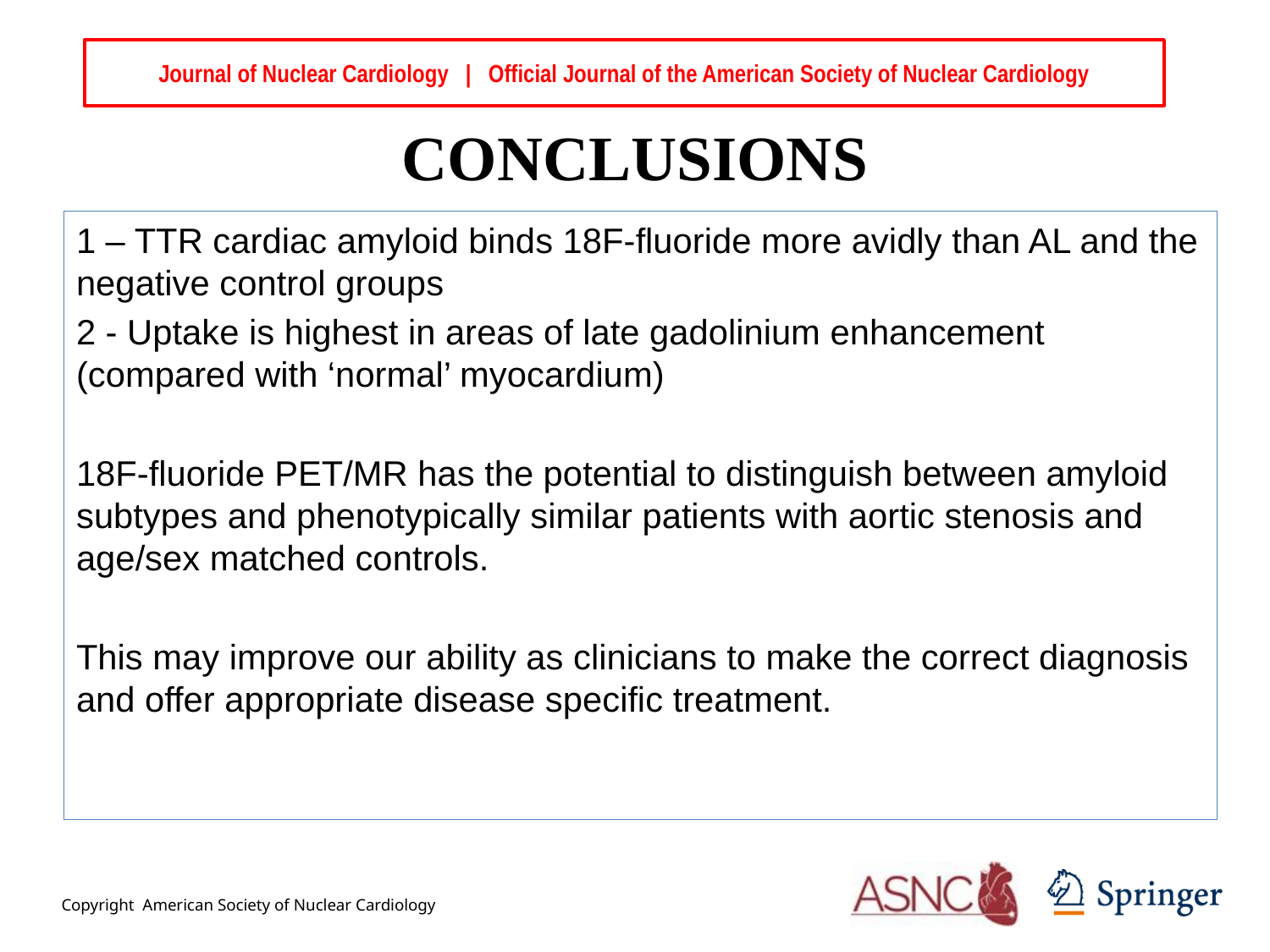

Journal of Nuclear Cardiology | Official Journal of the American Society of Nuclear Cardiology
# CONCLUSIONS
1 – TTR cardiac amyloid binds 18F-fluoride more avidly than AL and the negative control groups
2 - Uptake is highest in areas of late gadolinium enhancement (compared with ‘normal’ myocardium)
18F-fluoride PET/MR has the potential to distinguish between amyloid subtypes and phenotypically similar patients with aortic stenosis and age/sex matched controls.
This may improve our ability as clinicians to make the correct diagnosis and offer appropriate disease specific treatment.
Copyright American Society of Nuclear Cardiology
